# O3-8 A ban on smartphone usage during recess in increased 10-14 year old children's physical activity; a Danish school intervention study

**DOI:** 10.1093/eurpub/ckac094.024

**Published:** 2022-08-29

**Authors:** Charlotte Skau Pawlowski, Jonas Vesterggard Nielsen, Louise Stjerne Knudsen, Tanja Schmidt

**Affiliations:** Sports Science and Clinical Biomechanics, University of Southern Denmark, Odense M, Denmark

**Keywords:** mobile phone, intervention study, physical activity, policy, child health, SOPLAY, questionnaire

## Abstract

**Background:**

School recess provides a unique opportunity for children to be active. However, many children perceive smartphones as a key barrier for engaging in physical activity during recess. The aim was to investigate if a ban on smartphone usage during recess changed children's physical activity.

**Methods:**

During August-October 2020, children from grades 4-7 (10-14 yrs.) at six Danish schools were banned from using their smartphones during recess for a four-weeks period. Questionnaire and systematic observation (SOPLAY) data were collected from 814 children before intervention (baseline) and 828 during the last week of intervention (follow-up).

**Results:**

The mean frequency of physical activity significantly increased from baseline to follow-up (odds ratio = 1.370), as did physical activity on a moderate level (odds ratio = 1.387). Vigorous physical activity significantly decreased (odds ratio = 0.851). The increase in physical activity was found among both schools having outdoor and indoor recess, among both boys and girls and nearly equally among grades 4-7. Notably, we observed a much greater decrease in sedentary behavior and a slightly larger increase in moderate physical activity for girls than for boys and particularly boys decreased in vigorous physical activity. They might have changed their physical activity behavior in follow-up because more girls participated in physical activities, resulting in more children in the same space.

**Conclusion:**

This suggests that implementing a ban on smartphone usage during recess would, in line with the HEPA strategy, improve the everyday conditions for health among a broad range of children. To our knowledge, this is the first intervention study investigating if a ban on smartphone usage during recess changed schoolchildren's physical activity behavior. Thus, this study fills an important gap for researchers, school boards, teachers, health professionals, and politicians on how schoolchildren's physical activity during recess can be positively changed by policy.

